# Preparation of High-Performance Carbon Fiber-Reinforced Epoxy Composites by Compression Resin Transfer Molding

**DOI:** 10.3390/ma12010013

**Published:** 2018-12-20

**Authors:** Zeyu Sun, Jie Xiao, Lei Tao, Yuanping Wei, Shijie Wang, Hui Zhang, Shu Zhu, Muhuo Yu

**Affiliations:** 1 State Key Laboratory for Modification of Chemical Fibers and Polymer Materials, Donghua University, Shanghai 201620, China; sunzeyu@dhu.edu.cn (Z.S.); 1179137@mail.dhu.edu.cn (L.T.); sjwangdhu@126.com (S.W.); zhanghui@dhu.edu.cn (H.Z.); zhushu@dhu.edu.cn (S.Z.); 2 Key Laboratory of Shanghai City for Lightweight Composites, Donghua University, Shanghai 201620, China; 3 Research Center for Analysis and Measurement, Donghua University, Shanghai 201620, China; 4 Shanghai Engineering Research Center of Advanced Automotive Body Manufacturing, Donghua University, Shanghai 200092, China; yuanping.wei@ssdt.com.cn

**Keywords:** RTM, CM, CRTM, carbon-fiber reinforced epoxy composite, lightweight

## Abstract

To satisfy the light weight requirements of vehicles owing to the aggravation of environmental pollution, carbon-fiber (CF)-reinforced epoxy composites have been chosen as a substitute for traditional metal counterparts. Since the current processing methods such as resin transfer molding (RTM) and compression molding (CM) have many limitations, an integrated and optimal molding method needs to be developed. Herein, we prepared high-performance composites by an optimized molding method, namely compression resin transfer molding (CRTM), which combines the traditional RTM and CM selectively and comprehensively. Differential scanning calorimetry (DSC) and rotational rheometry were performed to optimize the molding parameters of CRTM. In addition, metallurgical microscopy test and mechanical tests were performed to evaluate the applicability of CRTM. The experimental results showed that the composites prepared by CRTM displayed superior mechanical properties than those of the composites prepared by RTM and CM. The composite prepared by CRTM showed up to 42.9%, 41.2%, 77.3%, and 5.3% increases in tensile strength, bending strength, interlaminar shear strength, and volume fraction, respectively, of the composites prepared by RTM. Meanwhile, the porosity decreased by 45.2 %.

## 1. Introduction

Lightweight automobiles are an effective way to realize energy conservation and emission reduction as vehicle usage increases. Carbon fiber (CF)-reinforced epoxy composites show excellent performances with light-weight, high-specific strength and stiffness, and excellent design-ability are a preferable alternative to replacing the traditional metal counterparts [1,2,3,4,5,6]. It has been widely used in aerospace, military products, lightweight automobiles, and so on [7,8,9,10]. The properties of composites depend on, not only the distribution and content of matrix and reinforcing materials, but also the molding methods. Composites comprising the same matrix and reinforcing materials may exhibit varying performances depending on the molding methods.

The resin transfer molding (RTM) method originated from the cold-casting process in the 1950s. Statistically, the product rate of RTM among developed countries has continued to increase by 10% in recent years [11]. In just 20 years from 1970 to 1990, the RTM process has become more mature and has been generally applied to manufacturing individual parts and even entire bodies [12]. RTM represents a closed-cavity molding technique that offers good dimensional precision and surface quality for the manufactured components. The ease of processing promotes wide applications of RTM in aerospace engineering [5]. As dry fibers are required to be fully impregnated during the injection process, the liquid resin for RTM should have a low viscosity and a wide injection temperature range [13,14]. The poor fluidity of epoxy resin seriously restricts its applications, and cracks and defects occur when epoxy-based components are subjected to external forces. Therefore, optimization of the RTM process is imperative in molding CF-reinforced epoxy composites to maintain its excellent performance [15,16]. Many studies have reported on the limitations of RTM technology. Feng et al. studied the formation of bubbles in the resin filling stage and proposed that the bubbles form at the end of the transverse fiber bundles [17]. Li et al. investigated the theory of defect formation, which could be used to predict the formation mechanisms of layer defects according to the flow pattern of resin [18]. Furthermore, Qin et al. believed that the ultrasonic treatment contributed to the improved impregnation of fiber reinforcements, thereby improving the mechanical properties of the composites [19]. 

Unlike RTM, CM (compression molding) is carried out under simultaneous application of heat and pressure. The large-scale heat regulation in CM is conducive to the fluidity of epoxy resin and impregnation of CFs in the mold cavity [20]. While the use of prepregs, which are impregnated with resin and curing agents, brought out problems in storage, transportation, high cost, and further restricted large-scale applications.

To solve the problem of complex molding conditions of RTM and CM in preparing CF-reinforced epoxy composites, some researchers reported compression-resin transfer molding (CRTM), which combined the RTM and CM selectively and optimally for the mass production of CF-reinforced composite automotive parts [21,22,23,24]. Bickerton and Kelly introduced the CRTM method computationally and theoretically, and carried out molding and analysis as well as experimental validation of numerical models applied to planar and non-planar geometries [23]. Sreekumar et al. studied the flow and heat transfer during the CRTM method [24]. Apart from the advantages of RTM and CM, the CRTM method enables efficient production and controllable production quality. 

Herein, we optimized the molding procedures and parameters of CRTM, and verified its excellent features by comparing it with RTM and CM. Firstly, resin was injected into the closed mold to ensure enough porosity of the fabric, and that the dry CFs were quickly impregnated under vacuum. Secondly, the excess resin was discharged under a low pressure to control the resin content. Thirdly, the pressure was raised to improve the compactness during the gelation stage. To confirm the optimum parameters of CRTM, the control variable method was used to prepare different composite samples by RTM and CM, respectively. Meanwhile optimum parameters of CRTM were obtained by debugging the molding parameters constantly. This study focused on investigating and optimizing the CRTM technology by using a double component epoxy as the reinforced matrix. The experiments showed that the CRTM technology meets the requirements of rapid batching, and therefore, its wide application in the industry is expected. 

## 2. Materials and Methods 

### 2.1. Materials and Characterizations

Bisphenol A-type epoxy resin (X4302A) and Isophorondiamine (C_10_H_12_N_2_, X4302B) as an amine curing agent were purchased from Bohui New Material Technology Co. LTD., Guangzhou, China. Plain weave CF cloth (T700-12k, Toray) was purchased from Lishuo Composite Materials Co. LTD., Shanghai, China. Prepreg (Plain weave T700-12k Toray/resin YPH-42T) was purchased from Huazheng Composite Materials Co. LTD., Shanghai, China. The curing agent of YPH-42T resin system was Dicyandiamide (C_2_H_4_N_4_). The release agent (19RSS) was purchased from Sino Composite Co. LTD., Shanghai, China. 

Equipment: Dual-component RTM injection equipment (ISOJET EQUIPEMENTS, Corbas, France); XLB-D350 plate vulcanization machine (Dehong Rubber and Plastic Machinery Co. LTD, Shanghai, China).

The rheological properties of the epoxy resin system were performed on the Physica MCR501 rheometer (Anton Paar, Graz, Austria) with 25-mm-diameter parallel plates under a nitrogen environment. 

The curing characteristics of resin system was carried out on the Modulated DSC2910 differential scanning calorimeter (TA Instruments, New Castle, DE, USA) equipment. The heating rate was set as 5 °C/min, 10 °C/min, 15 °C/min and 20 °C/min under nitrogen atmosphere with a flowing rate of 50 mL/min to record the curing exotherm of resin system.

The glass transition temperature of resin casting body was carried out on the Modulated DSC2910 differential scanning calorimeter (TA Instruments, New Castle, DE, USA) at the heating rate of 10 °C/min under nitrogen atmosphere.

The tensile performance test of the composite samples was performed on the universal testing machine (LABSANS Material Testing Co. LTD, Shenzhen, China) at the stretching velocity of 2 mm/min according to ASTM D3039. Five pieces of specimen were tested to get the average value of the result as the tensile strength. The size of the specimen was 250 × 25 × 2.5 mm^3^. 

The compressive performance test of the composite samples was performed on the universal testing machine at the loading rate of 1.3 mm/min according to ASTM D6641. Five pieces of specimen were tested to get the average value of the result as the compressive strength. The size of specimen was 140 × 12 × 4 mm^3^. 

The bending performance test of the composite samples was performed on the universal testing machine at the testing speed of 1.0 mm/min according to ASTM D7264. Five pieces of specimen were tested to get the average value of the result as the bending strength. The size of specimen was 155 × 13 × 4 mm^3^. 

The interlaminar shear performance test of the composite samples was performed on the universal testing machine at the testing speed of 1.0 mm/min according to ASTM D2344. Five pieces of specimen were tested to get the average value of the result as the interlaminar shear strength. The size of specimen was 12×4×2 mm^3^. 

The metallographic microscopy test was performed on the Axiovert 40 MAT microscope (Carl Zeiss AG, Oberkochen, Germany), the prepared composite samples were polished first with sandpaper and then with metallographic sandpaper. The samples were then scanned to observe the interface of the fiber and resin system, and the internal pore size and distribution after a final polish with the instrument.

The fiber volume and porosity tests were performed according to the GB/T 3365-2008 CF-reinforced plastic fiber volume content and porosity test standard.

### 2.2. Preparation of Casting Body by Bisphenol A-Type Epoxy Resin System

**Figure 1:**
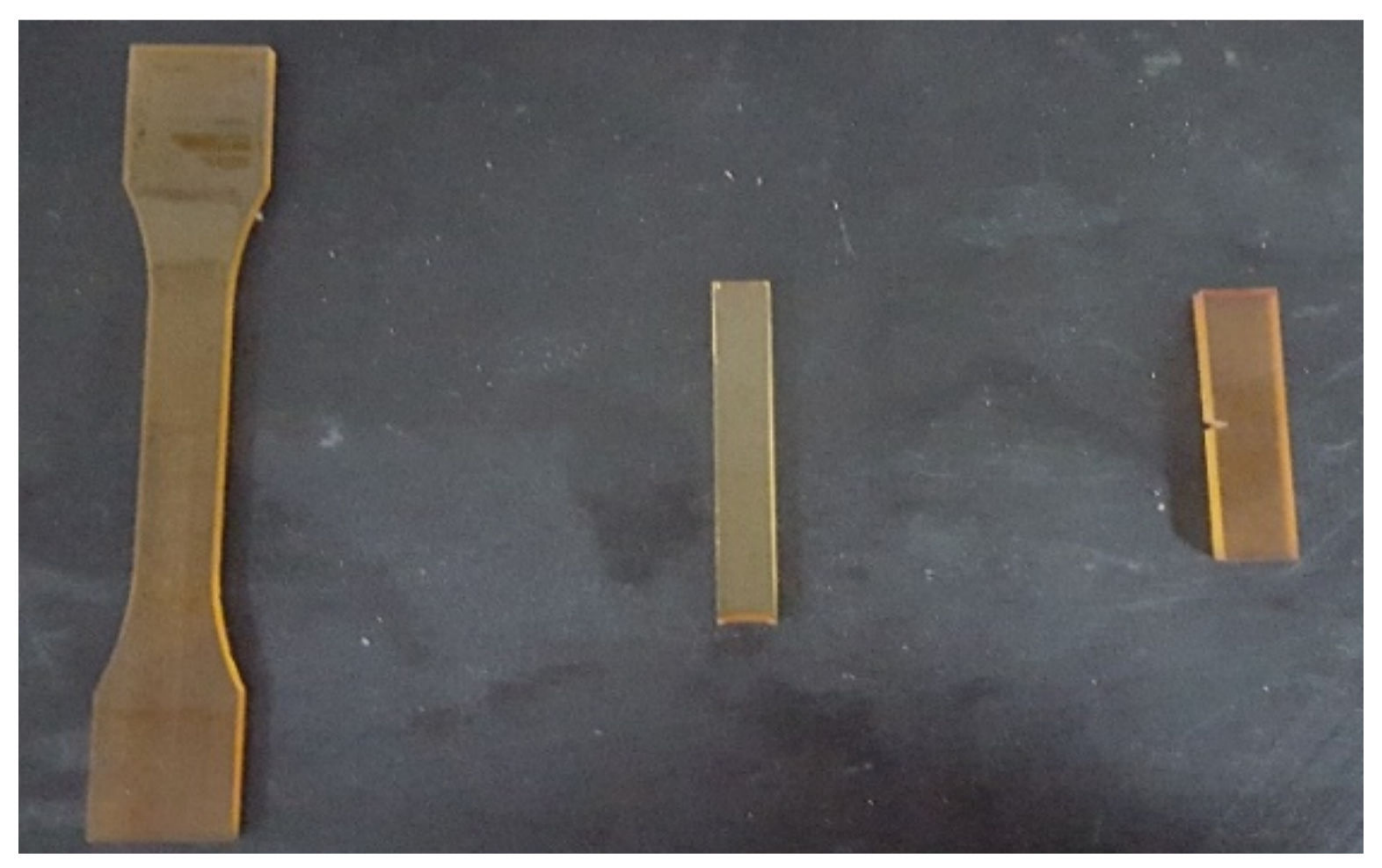
Photo of resin casting body.

The bisphenol A-type epoxy resin was blended with the amine curing agent in a mass ratio of 100:45 and then vacuum degassed for 30 min at 40 °C. Meanwhile, the mold was preheated in a vacuum oven at 40 °C. After degassing, the resin was injected into the mold slowly and then solidified in the vacuum oven to obtain a resin casting body as shown in Figure 1.

### 2.3. Preparation of Composite Samples by CM

**Scheme 1:**
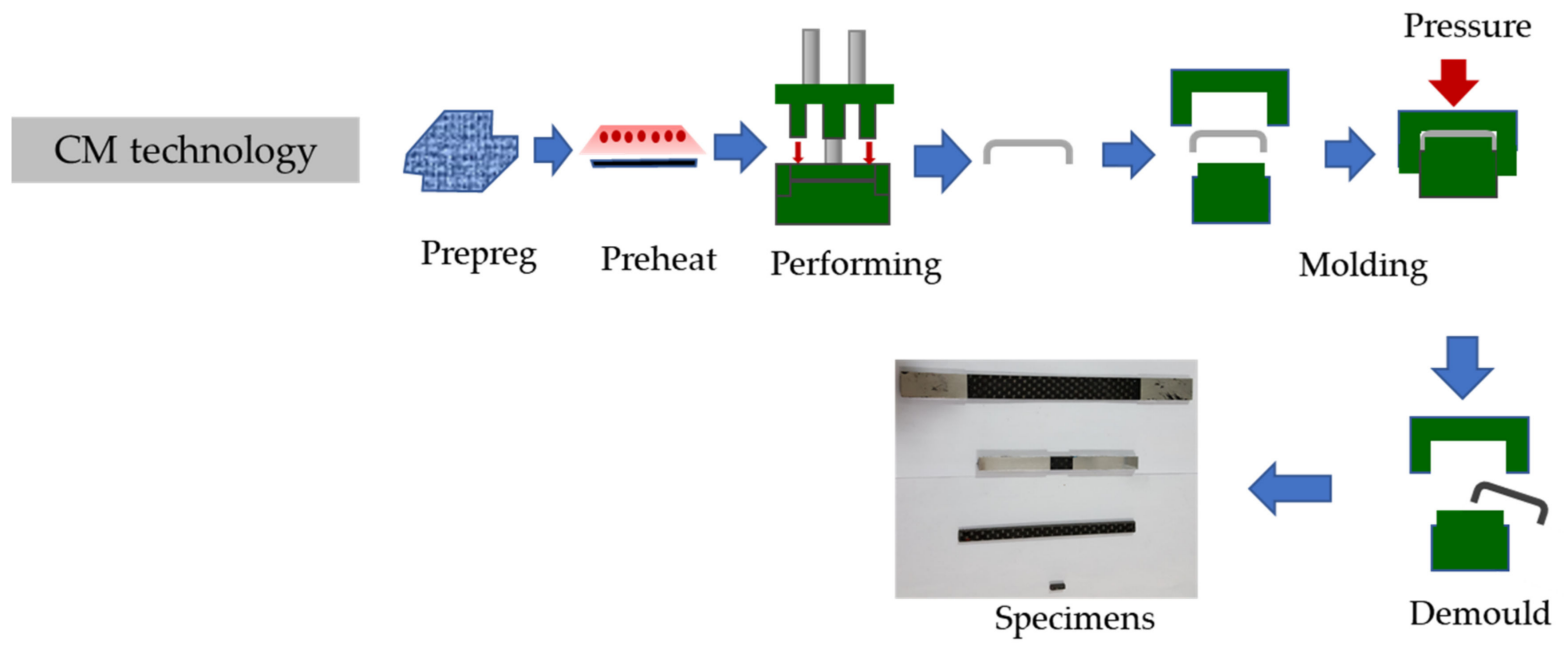
Flow chart of compression molding (CM) technology.

Prepreg was used to achieve the optimum processing parameters by the control variable method. By comparing the surface morphology and mechanical properties of the composite samples, the optimum parameters of CM were confirmed. The molding scheme and specimens’ photo of CM technology are shown in Scheme 1.

### 2.4. Preparation of Composite Samples by RTM

**Scheme 2:**
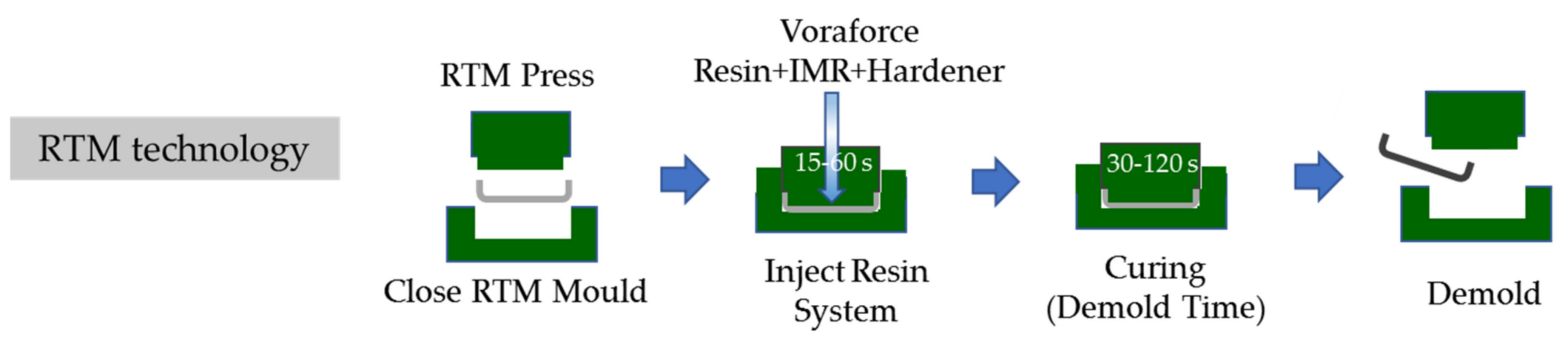
Flow chart of resign transfer molding (RTM) technology.

A certain number of layers of plain CF dry clothes were gradually laid in the mold and the mold was closed. The RTM equipment was turned on and RTM was performed with specific RTM parameters. The sprue gate valve was closed when the resin system filled the cavity. The exhaust port valve was closed when little bubbles appeared in the discharged resin system from one end of the exhaust port. Then, the composite was cured in an oven under specific conditions and ejected from the mold after cooling. The RTM scheme is shown in Scheme 2.

### 2.5. Preparation of Composite Samples by CRTM

**Scheme 3:**
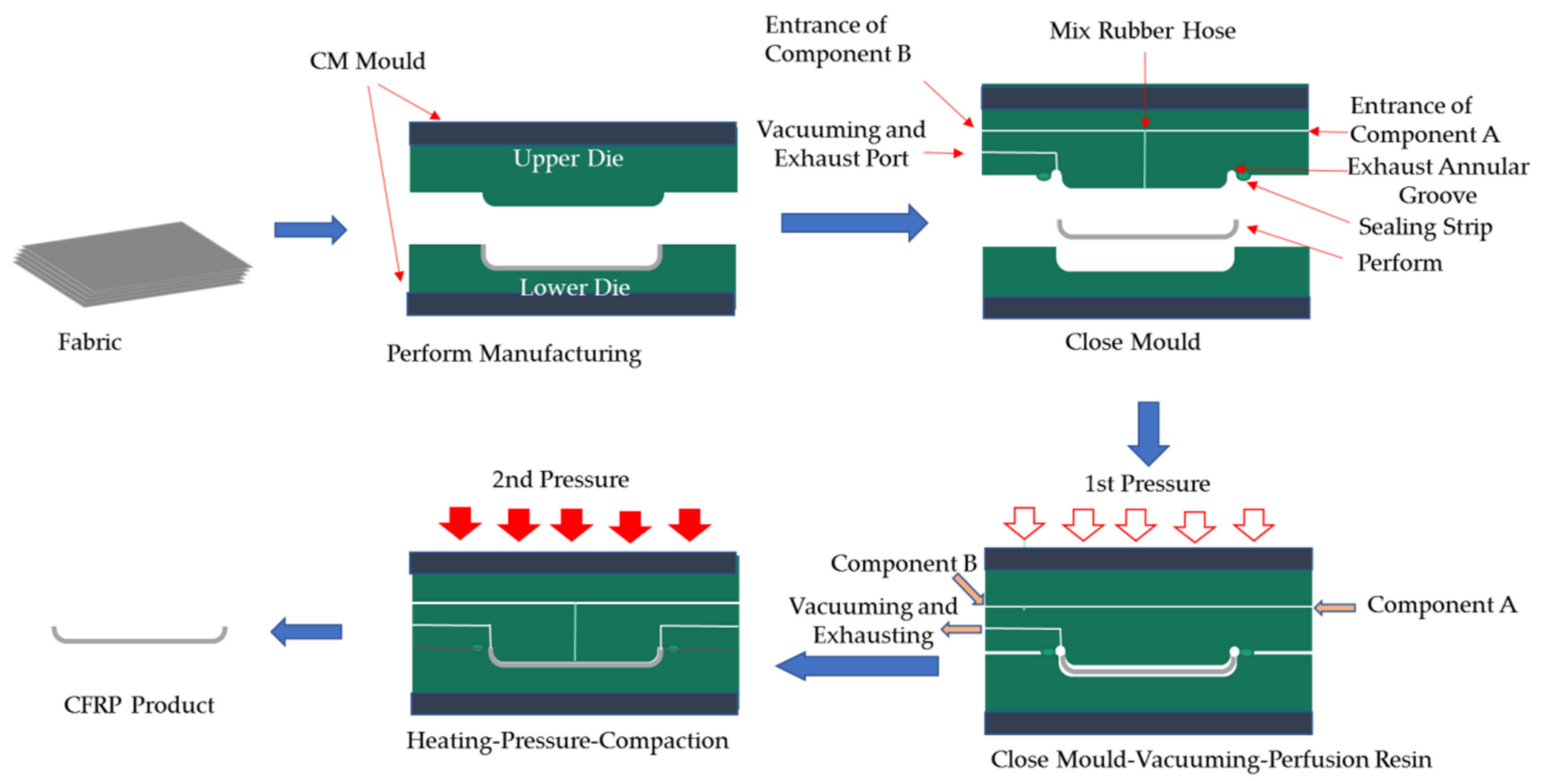
Flow chart of compression-resin transfer molding (CRTM) technology.

A certain number of layers of plain CF dry clothes were gradually laid in the mold and the mold closure was controlled to ensure high permeability. The resin was injected according to specific parameters in vacuum. The sprue gate valve was closed when the resin system filled the cavity. The exhaust port valve was closed when little bubbles appeared in the discharged resin system from one end of the exhaust port. The mold was closed to a certain degree to improve the resin content and impregnation. A certain pressure was applied under heating to discharge residual bubbles. The composite was heated to the curing temperature to improve the compactness of the materials under certain pressure according to the CM technology. The composite was ejected from the mold after cooling. The CRTM scheme is shown in Scheme 3.

## 3. Results and Discussions

### 3.1. Properties of Epoxy Resin System

#### 3.1.1. Thermal Properties of Epoxy Resin System

Composite materials should withstand elevated temperatures to maintain structural integrity. If the service temperature exceeds the glass transition temperature (T_g_), the polymer matrices will rapidly lose their strength and stiffness, which in turn will weaken the fiber-matrix interfacial bonding strength [10,25]. As a result, the mechanical properties may drastically deteriorate at elevated temperatures, leading to a breakdown in structural integrity. To maintain structural integrity, it is critical to investigate the thermal characteristics of the epoxy resin in the molded CF-reinforced composites. 

**Figure 2:**
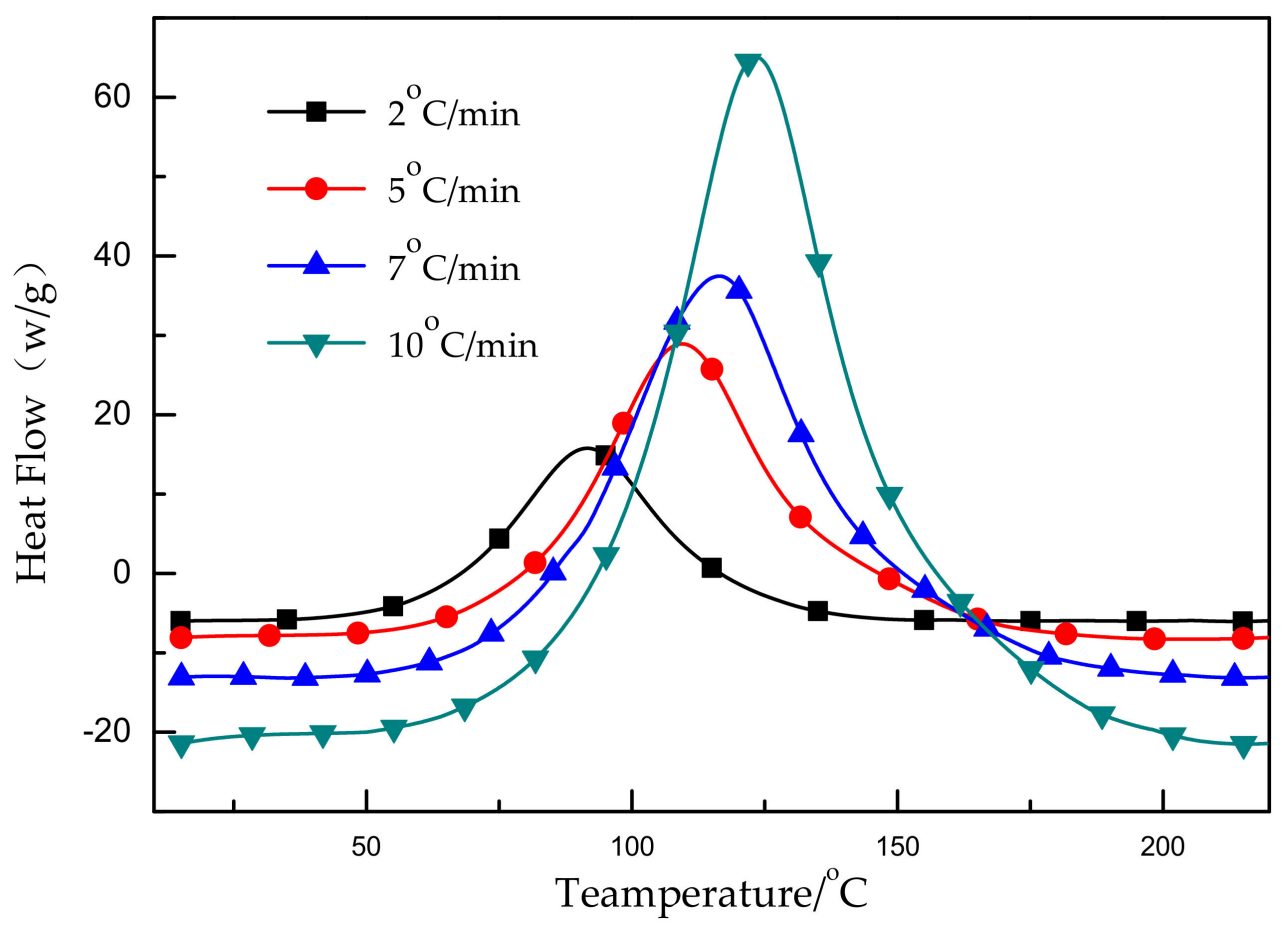
Differential scanning calorimetry (DSC) curves of the resin system.

The differential scanning calorimetry curves of the resin system at different heating rates are displayed in Figure 2. During the curing reaction, the initial temperature (T_i_), peak temperature (T_p_), and finial temperature (T_f_) increased as heating rate increased, as summarized in Table 1. 

**Figure 3:**
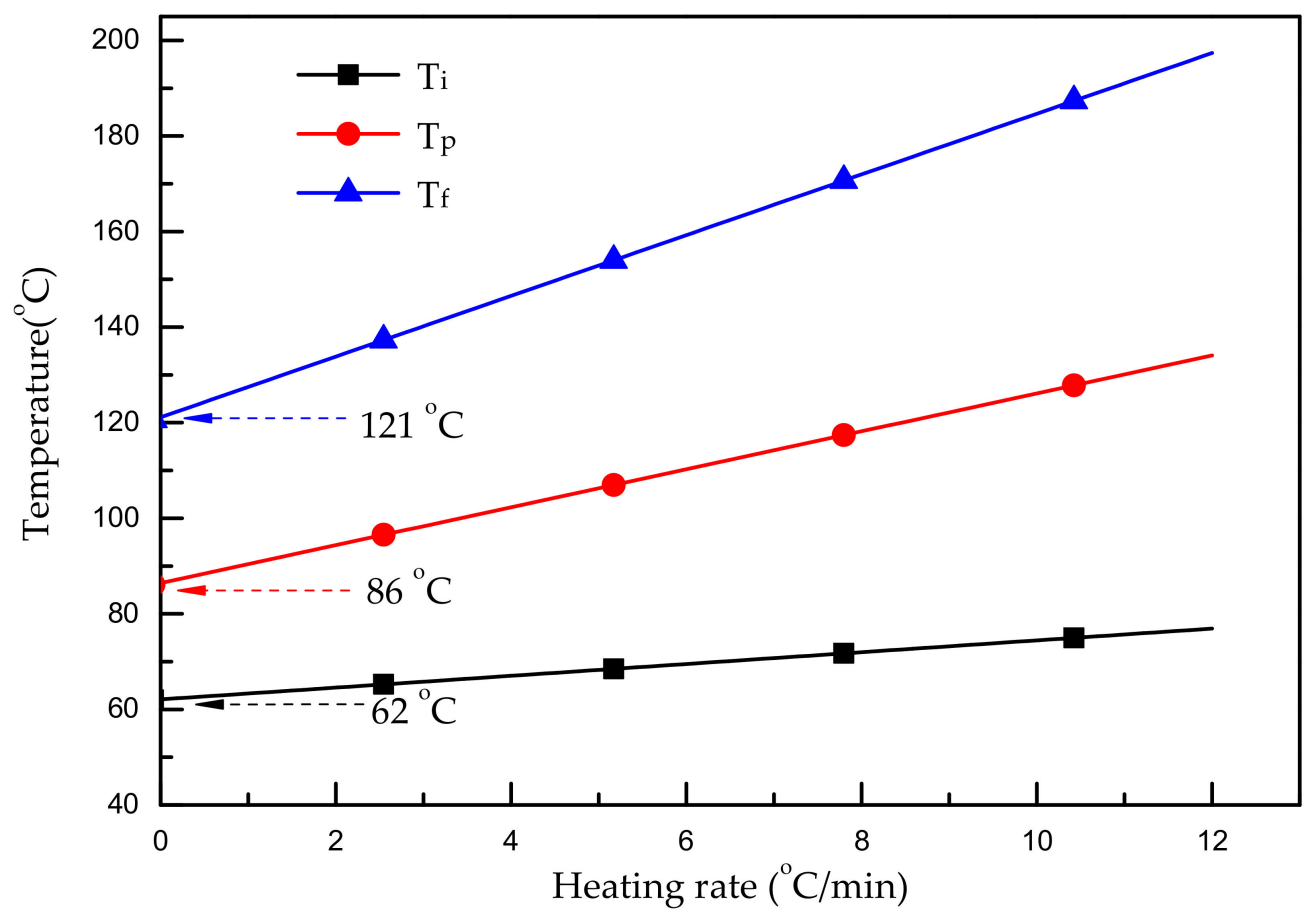
Extrapolation method to determine curing temperature of the resin system.

Based on the characteristic temperatures at different heating rates listed in Table 1, the characteristic temperatures at a heating rate of 0 °C/min were calculated by the extrapolation method. As shown in Figure 3, the Y-intercept values are the characteristic temperature values including T_i_, T_p_ and T_f_, which are 62 °C, 86 °C, and 121 °C, respectively. It could be concluded that the crosslinking temperature was about 90 °C and the curing temperature was around 120 °C.

**Figure 4:**
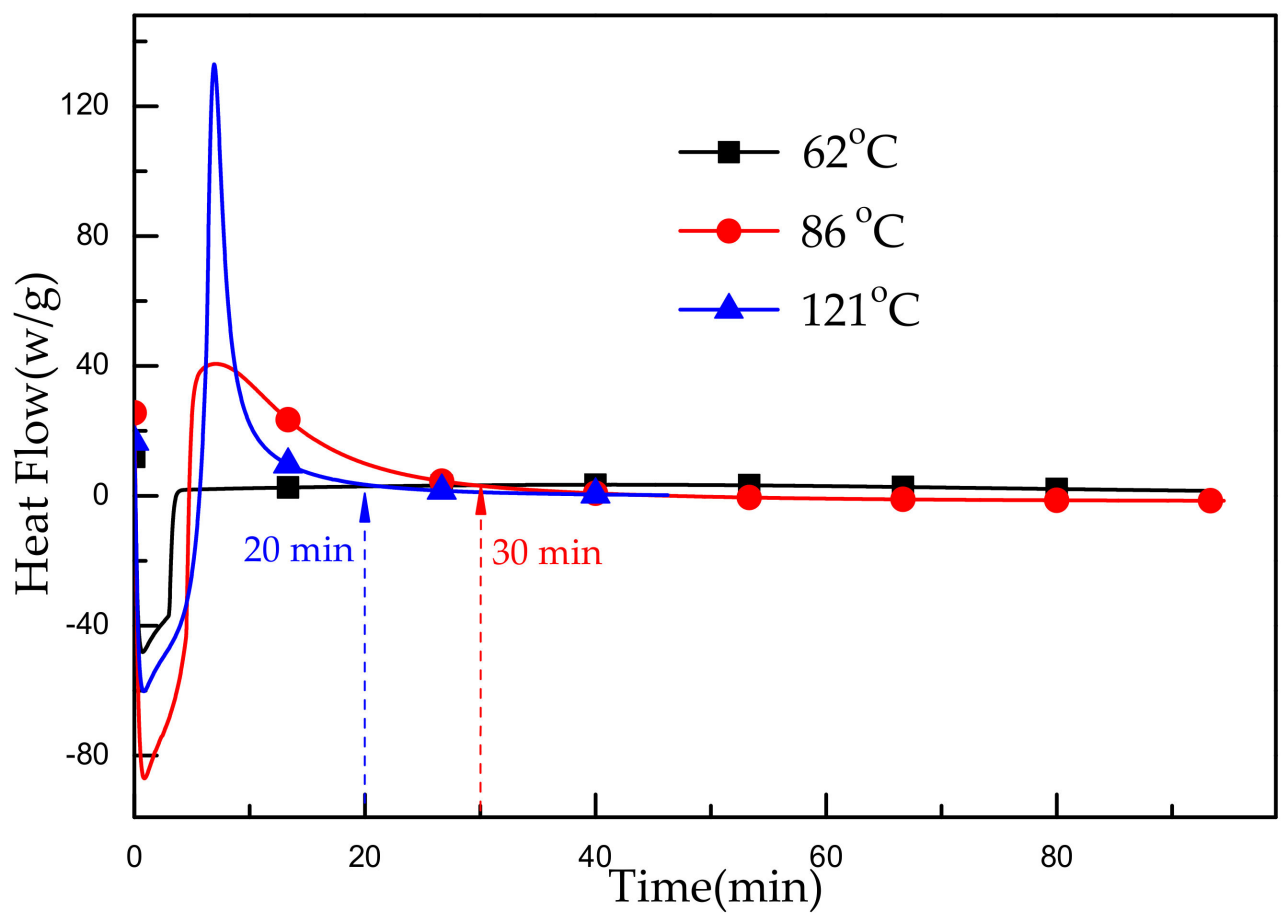
Isothermal DSC curves of the resin systems.

The characteristic temperatures of the X4302A/B resin system were calculated by the extrapolation method from the DSC curves of the resin system, which were obtained by the DSC measurement. As shown in Figure 4, at characteristic temperatures of 62 °C, 86 °C and 121 °C, the heat release time under a constant temperature was 90 min, 30 min, and 20 min, respectively. Theoretically, the post-curing temperature and time, play important roles in the curing stage of the resin system. A proper extension of time contributed to an improved crosslinking degree, which could improve the mechanical properties to some extent. The experiment confirmed that the curing conditions of X4302A/B resin was set as 90 °C/30 min +120 °C/60 min. 

#### 3.1.2. Rheological Properties of Epoxy Resin System

**Figure 5:**
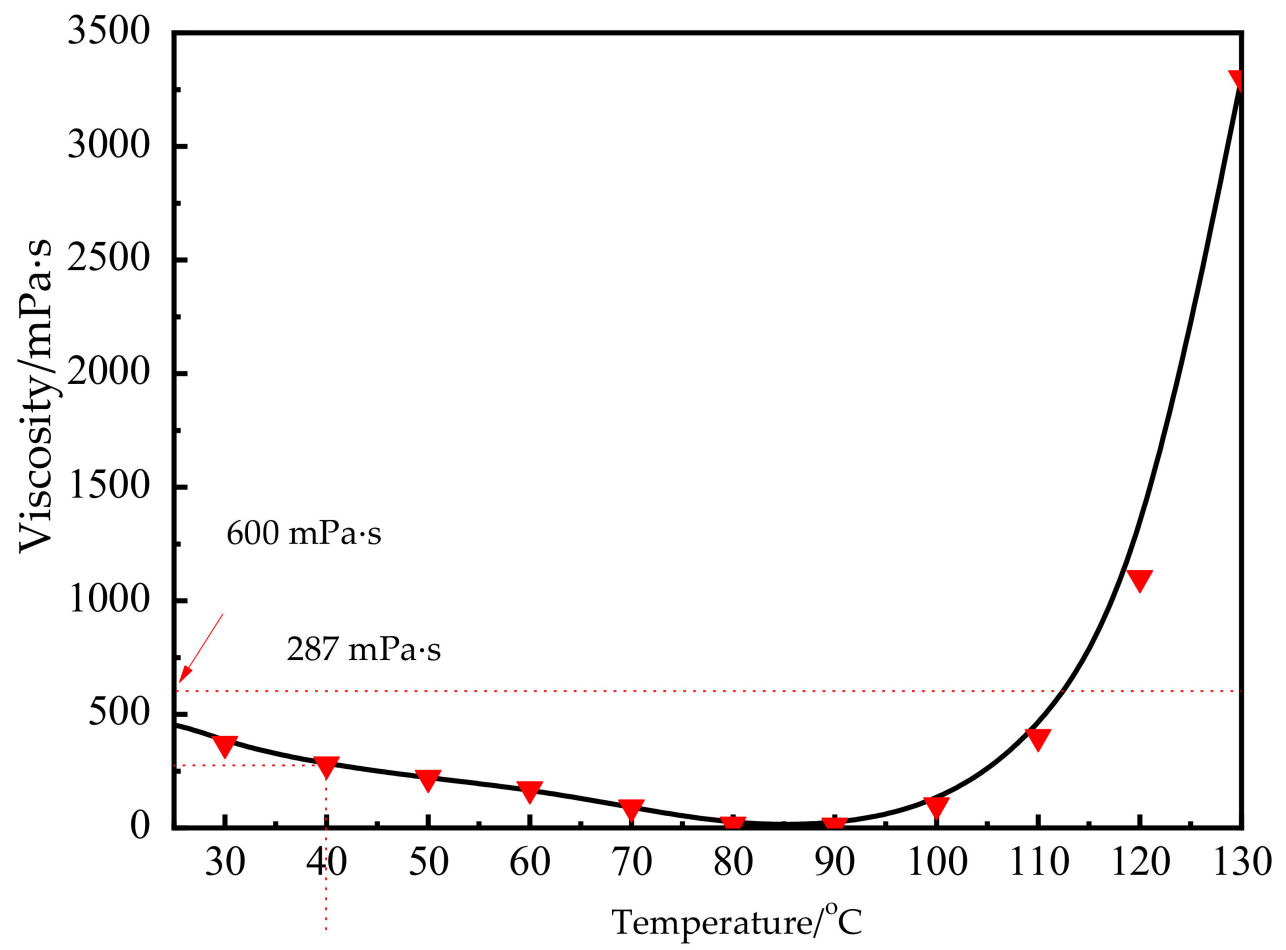
Viscosity variations of X4302A/B resin system with temperature.

The rheological properties of the epoxy resin system include viscosity and elasticity. To achieve complete impregnation, a lower viscosity of the resin before the gel point is essential. Ordinally, the viscosity value of RTM technology should be below 600 mPa·s. Owing to the insignificant influence of shear rate on the rheological properties before the gel point [26], the change in resin viscosity is mainly associated with an increase in temperature. As shown in Figure 5, the viscosity of the resin system decreased slowly and then increased sharply with increasing temperature. From 40 °C to 90 °C, the viscosity values met the requirements of RTM. A low-viscosity platform appeared around 85 °C and reached the minimum value, which contributed to good impregnation of the CFs by the resin system. Furthermore, the resin system began to cure at 60 °C, as shown in Figure 4, and the viscosity of the resin below 40 °C reached 287 mPa·s, which satisfied the viscosity requirement of molding. Therefore, 40 °C was chosen as the optimal injection temperature. 

Mechanical properties of the resin casting body performed on the universal testing machine were listed in Table 2. The high strength and modulus lay the foundation for the carbon fiber reinforced composites.

### 3.2. Mechanical Properties of Composite Prepared by CM

To compare the mechanical properties of the composites prepared by CM, RTM, and CRTM, a common prepreg was used for CM (fabric /T700-12k, resin/YPH-42T) and shows excellent properties, while a dry CF cloth was used for RTM and CRTM.

**Figure 6:**
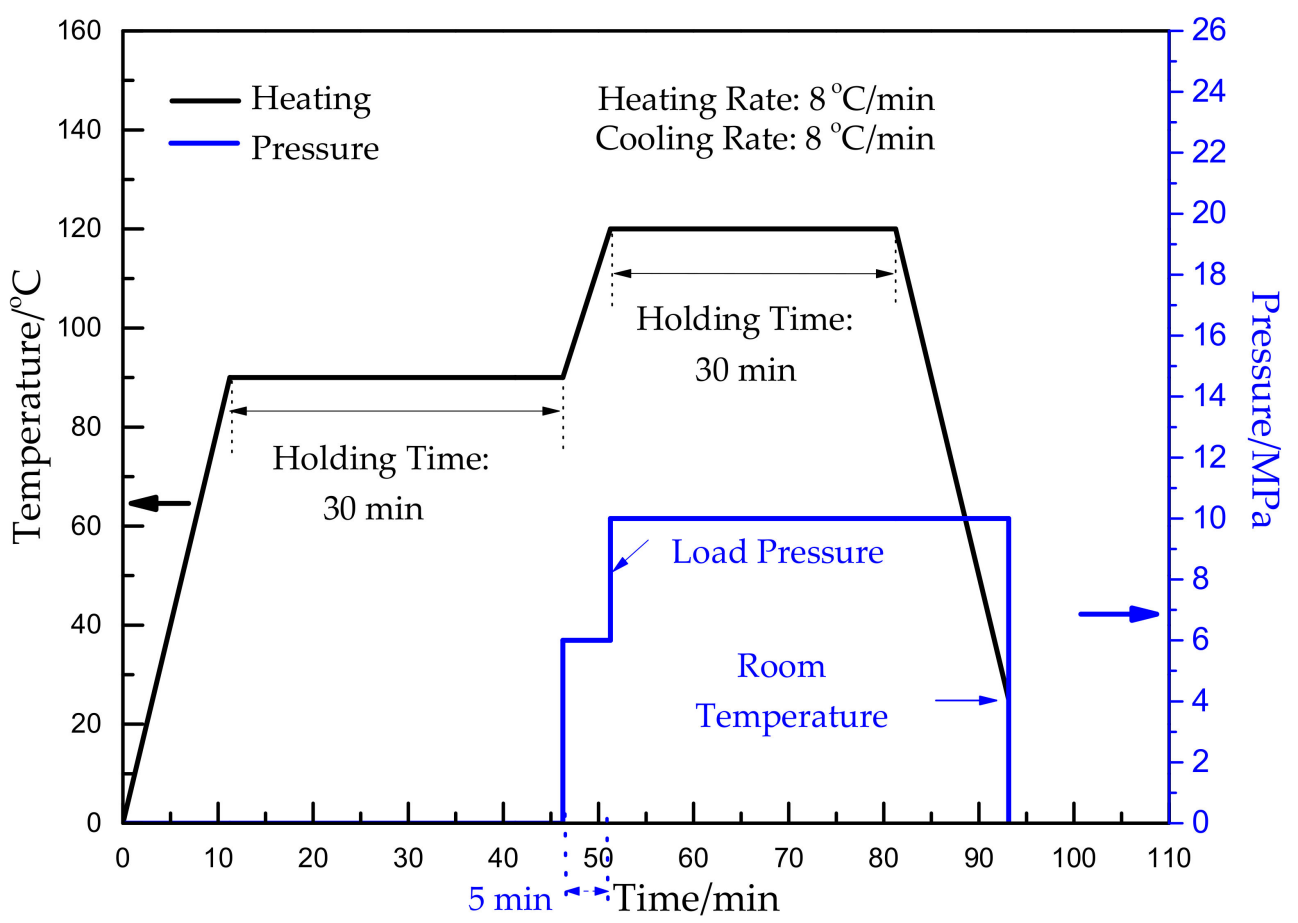
Subsection heating and pressurization schemes of the molding process.

The pressure duration played an important role in the molding process. It could accelerate the impregnation of the CFs by the resin during the low-viscosity stage of the resins, which is beneficial to the exclusion of bubbles present between the filaments and the combination of resin with the reinforcement. Herein, the subsection heating plan shown in Figure 6 was set as the optimal heating and pressing solution. 

**Figure 7:**
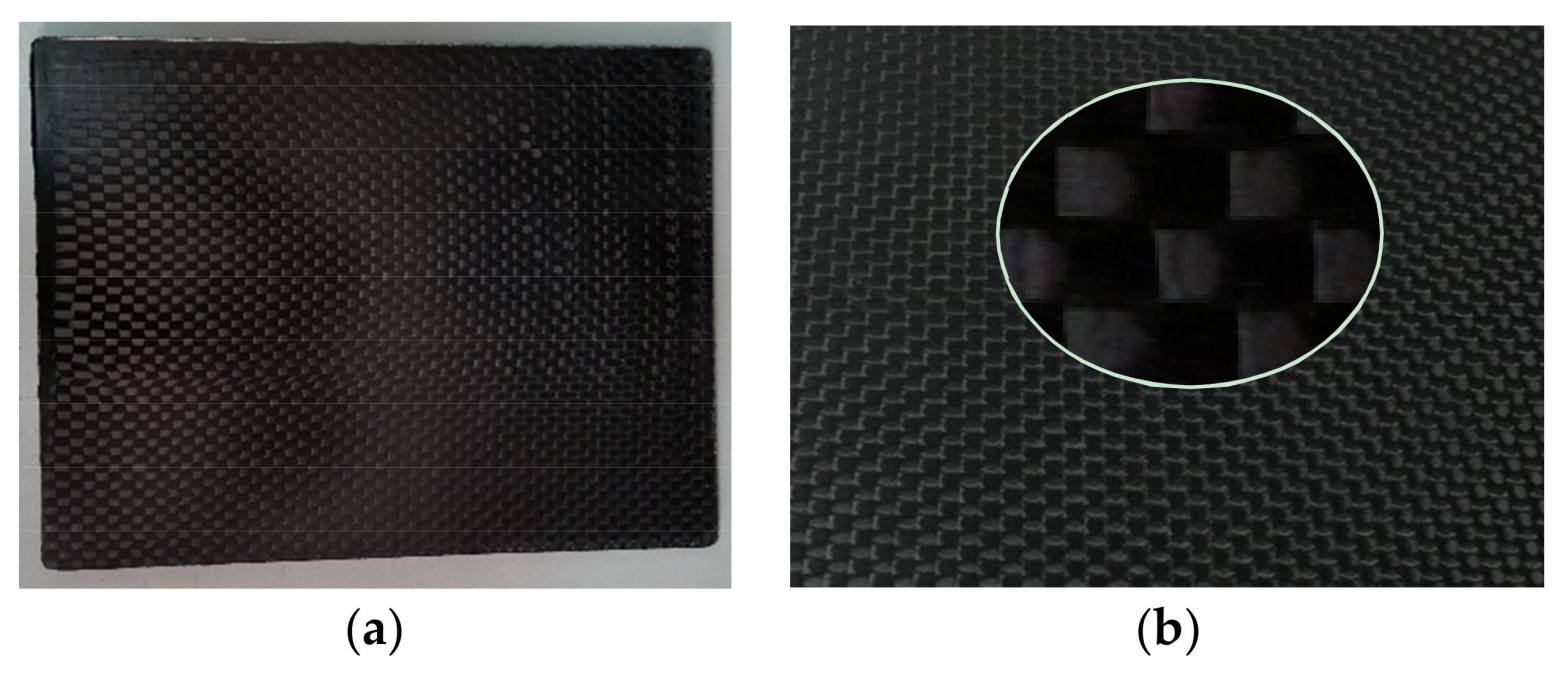
Specimen’s photo (**a**) and surface morphology contrast (**b**) of the composites prepared by CM process.

A composite sample molded by the CM method is shown in Figure 7. The sample shows a smooth surface without any blowholes, which verified the feasibility of the heating and pressure plan. 

The measured mechanical properties and CV (coefficient of variance) values of the composite samples are displayed in Table 3.

### 3.3. Mechanical Properties of Composite Samples Prepared by RTM

During the RTM process, a dry carbon cloth was used as the CF source. After 30 min of vacuuming, the bisphenol A-type epoxy resin and amine curing agent were poured from a resin tank and curing agent tank, respectively, and mixed at the injection port to inject into the mold at a certain injection rate. Thus, the resin temperature, infusion speed, injection pressure, and positions and numbers of sprue gate and exhaust port play important roles in composite preparation. Based on the thermodynamic and rheological analyses of resin combined with experimental results, the parameters of the RTM process are listed in Table 4.

**Figure 8:**
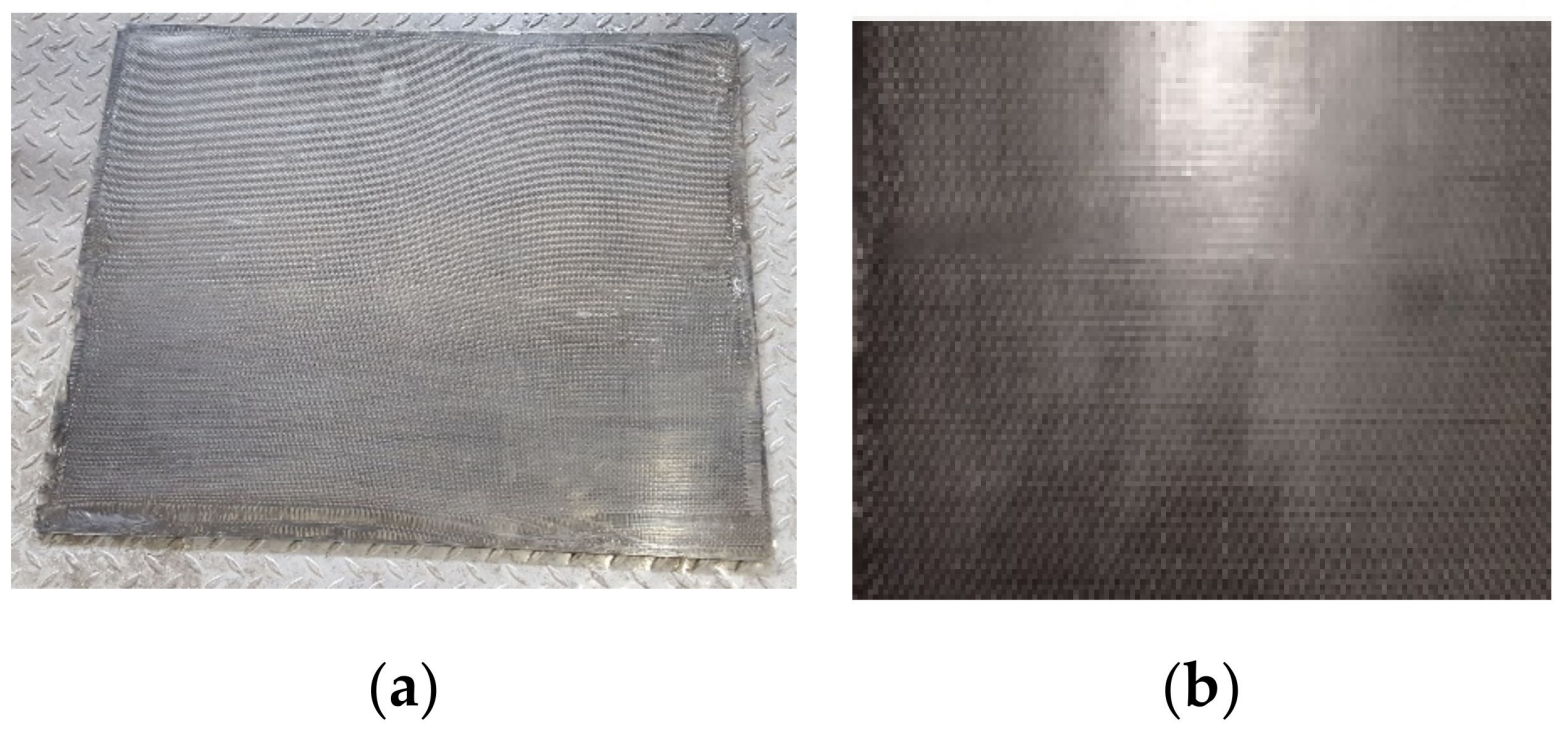
Specimen’s photo (**a**) and appearance of template (**b**) prepared by RTM process.

As shown in Figure 8, the composite sample appears much smoother with no dry spot or blowhole. The mechanical properties and CV (coefficient of variance) values of the composite samples are summarized in Table 5.

### 3.4. Preparation and Mechanical Properties of Composite Samples Prepared by CRTM

**Figure 9:**
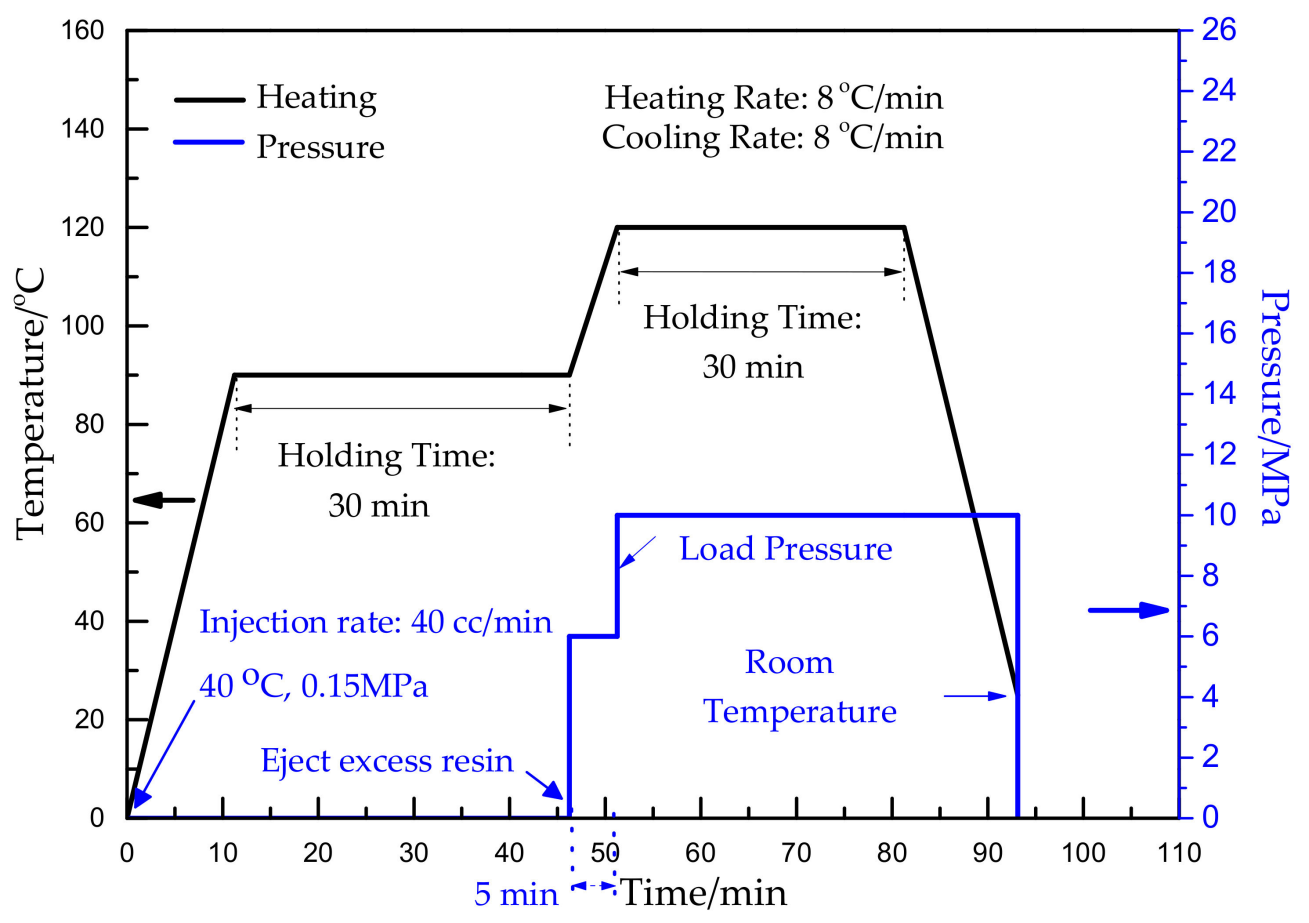
Subsection heating and pressurization schemes of the molding process.

By investigating the optimum parameters of CM and RTM, we obtained the optimum parameters of CRTM. Firstly, during the injection stage, the injection pressure was set as 0.15 MPa at a rate of 40 cc/min under 40 °C. Secondly, during the curing stage of CM, the sample was heated up to 90 °C under no pressure and held for 30 min. Thirdly, a pressure of 6 MPa was applied for 5 min to discharge the excess resin and bubbles in the mold cavity. Finally, load pressure and an additional load pressure of 10 MPa was applied with heating up to 120 °C for 60 min. The subsection heating and pressure plan is shown in Figure 9.

**Figure 10:**
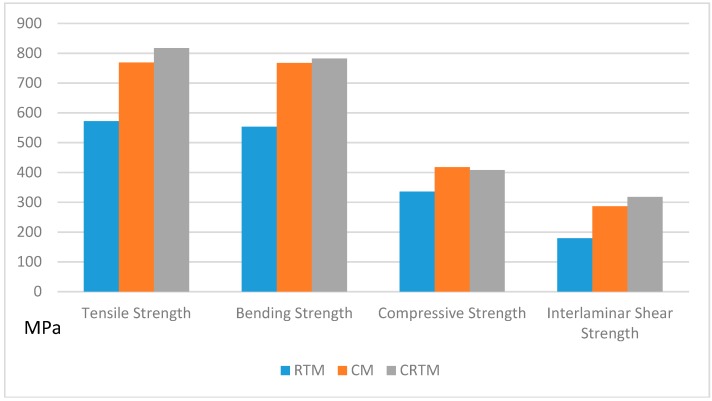
Mechanical properties of samples prepared by CM, RTM, and CRTM.

The mechanical properties and CV values of the composite samples prepared by CRTM are listed in Table 6. The mechanical properties of the samples prepared by CRTM are superior to those of the samples prepared by CM and RTM, as shown in Figure 10. The tensile strength, bending strength, compressive strength, and interlaminar shear strength improved by 42.9%, 41.2%, 21.5%, and 77.3%, respectively, compared with those of the samples prepared by RTM. 

**Figure 11:**
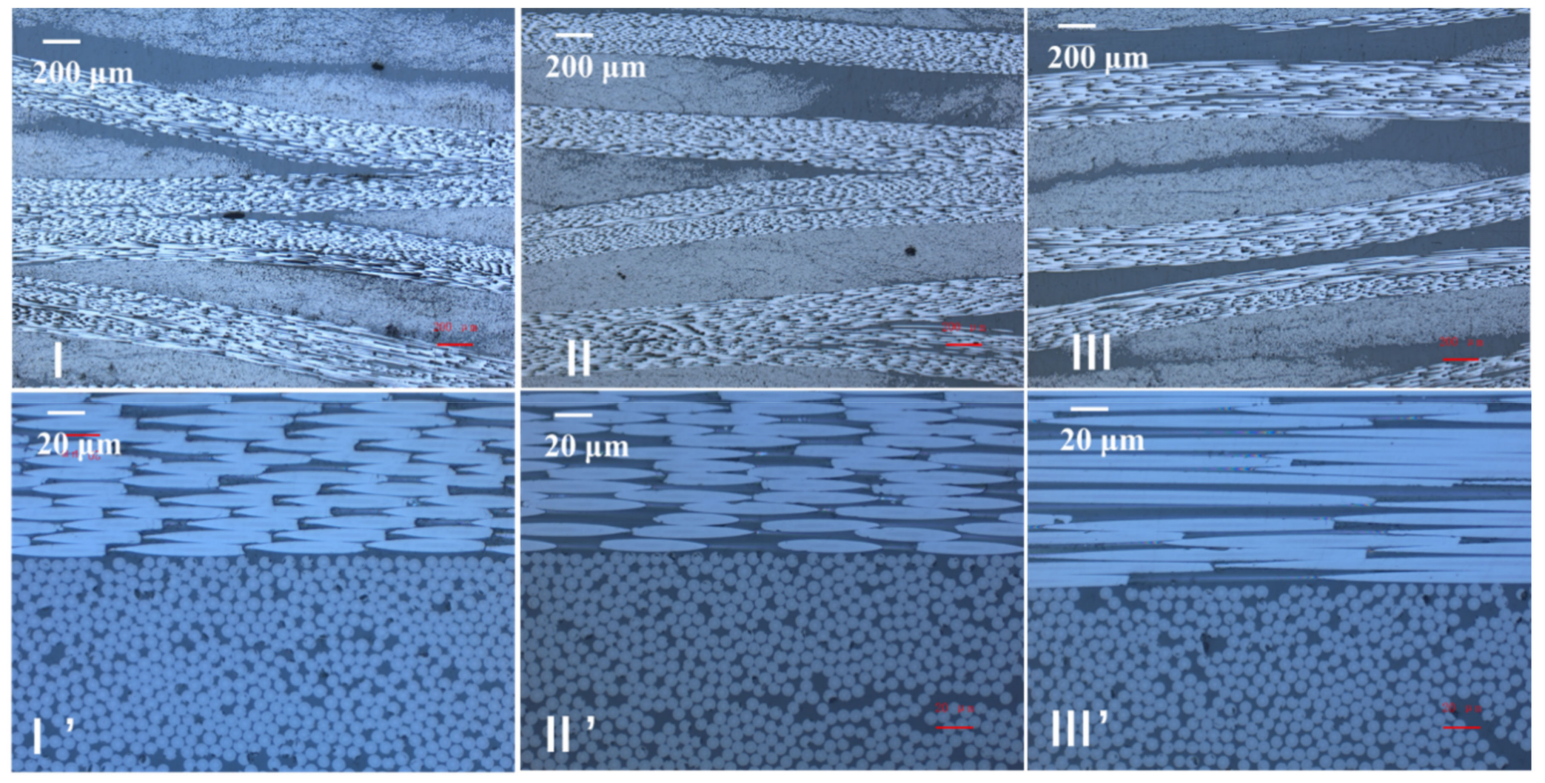
Microstructure of composite samples prepared by different molding methods: (I, II, and III) fiber equatorial plane section (scale: 200 μm) and (I’, II’, and III’) fiber axial section (scale: 20 μm) of composites prepared by CRTM, CM, and RTM, respectively.

Metallurgical microscopy photos of the composite samples prepared by the three molding methods are shown in Figure 11. Here, images I, II, and III represent the fiber equatorial plane section and images I’, II’, and III’ represent the fiber axial section of the composites prepared by CRTM, CM, and RTM, respectively. In the equatorial plane section of I (CRTM) and II (CM), the CFs are distributed uniformly in the resin, while a resin-enriched area is seen in III (RTM). In the fiber axial section images, the inter-layer fibers appear more aligned and compact and the inter-layer resin system content is minimal. 

The volume fraction and porosity of the composites are summarized in Table 7. The volume fraction of the composite prepared by CRTM improved by 5.3% and the porosity decreased by 45.2% compared with those of the composite prepared by the RTM process; this demonstrates the optimal processing conditions of CRTM.

## 4. Conclusions

In this study, carbon fiber-reinforced epoxy composites with high-performance were prepared by CRTM technology. Light-spots of CRTM technology were concluded as,

(1) the molding parameters of CRTM technology was confirmed by investigation on influence factors of CM and RTM technology, including filling temperature, infusion speed, heating and pressure scheme, etc.
(2) the experimental results showed that the composites prepared by CRTM exhibited superior mechanical properties than those of the composites prepared by RTM and CM. The composite prepared by CRTM showed up to 42.9%, 41.2%, 77.3%, and 5.3% increases in tensile strength, bending strength, interlaminar shear strength, and volume fraction, respectively, that those of the composites prepared by RTM. Meanwhile, the porosity decreased by 45.2 %.
(3) CRTM technology saves cost in molding carbon fiber reinforced composites compared with CM technology, which avoids the problems of storage, transportation, and high cost of prepreg.

In conclusion, the CRTM method has great potential for mass production and could be used for molding structural components of automobiles with high productivity, low cost, and excellent properties.

## Figures and Tables

**Table 1:** Characteristic temperatures of the resin system.

Heating Rate (°C/min)	T_i_ (°C)	T_p_ (°C)	T_f_ (°C)
2	64	94	133
5	68	106	153
7	71	114	165
10	74	126	184
0	62	86	121

**Table 2:** Mechanical properties of resin casting body.

Tensile Strength /MPa	CV /%	Tensile Modulus /GPa	Elongation /%	Bending Strength /MPa	CV /%	Bending Modulus /GPa	Impact Strength/KJ/m^2^	CV /%
768.56	3.46	3.01	4.02	767.64	2.82	3.07	16.87	4.66

**Table 3:** Mechanical properties of composite sample molded by compression molding (CM).

Tensile Strength /MPa	CV /%	Bending Strength /MPa	CV /%	Compressive Strength /MPa	CV /%	Interlaminar Shear Strength /MPa	CV /%
768.56	2.82	767.64	3.86	417.59	4.48	286.90	4.22

**Table 4:** Parameters of resign transfer molding (RTM) process.

Injection Rate/cc·min^−1^	Injection Pressure/MPa	Temperature/°C
40	0.15	40

**Table 5:** Mechanical properties of samples under different conditions.

Tensile Strength /MPa	CV /%	Bending Strength /MPa	CV /%	Compressive Strength /MPa	CV /%	Interlaminar Shear Strength /MPa	CV /%
571.69	3.24	553.59	4.32	335.46	3.66	179.05	2.98

**Table 6:** Mechanical properties of composite samples prepared by compression resin transfer molding (CRTM).

Tensile Strength /MPa	CV /%	Bending Strength /MPa	CV /%	Compressive Strength /MPa	CV /%	Interlaminar Shear Strength /MPa	CV /%
817.51	3.67	781.90	4.05	407.54	4.12	317.53	3.89

**Table 7:** Volume fraction and porosity of composite samples prepared by CRTM, RTM, and CM.

Processing Style	Volume Fraction/%	Porosity/%
CRTM	78.1	0.23
CM	78.5	0.26
RTM	74.2	0.42
